# 

**Published:** 1872-01

**Authors:** 


### INDEX OF SUBJECTS.

### ORIGINAL COMMUNICATIONS.

Artesian Wells........................................................................ 1
A Case of Paralysis of Mortor Portion of 7th Cranial Nerve.................................10
Foreign Correspondence.....................................................................11

### EXTRACTS AND GLEANINGS.

On some Preparations of Iron, 17; Chloral Hydrate in Infantile Convulsions—Puerperal Convulsions Successfully Treated without Bleeding, 19; Kneading in Constipation—Improved Dover's Powder—Hooping-Cough—Oxaluria, 21; Amenorrhœa and Dysmenorrhœa—Scarlatina—Infantile Wasting, 22; Collodion in Chilblains—Treatment of Psoriasis—Dysentery, 23; Pityriasis Versicola—Oxalates in the Urine—Treatment of Tympany by Lobelia, 24; Sympathetic Nervous Trouble from the Presence of a Tenia—Frozen Beef-Essence, 25; Bromide of Sodium—Turpentine in Ring-worm, 26; Deterioration of Milk in Feeding-Bottles—Nauseous Medicines, 27; Hypertrophy of Os Uteri Treated by Puncture, 28; Dyspepsia, Constipation—Gleet—Iodide of Starch Poultice, 29; Infantile Remittent Fever—Tincture of Veratrum in Pneumonia of Children, 30; Malignant Small-Pox treated with Sulphur Fumigations—Treatment of Capillary Bronchitis in Children by Warm Vapor, 31; Uuwholesome Candies—Ulcerated Sore Throat, 32; Method of Promptly Relieving Facial Dental Neuralgia—Aconite to Children, 33; To Expell Foreign Bodies from the Larynx—Chloral to Infants—Diptheria, 34; Bloody Discharges After Micurition—Diebetes—Irritation of Stomach, 35; Treatment of Cholera Infantum—Aphasia, 36; Inunction in Diseases of Children—Cases of Palpitation of the Heart, 37; Epithelima Removed by Bromide, 38; A Case of Calculus Nephritis—Puerpetral Convulsions, 39; The Use of Earth as a Dressing in Severe Burns—The Use of Acetic Acid in Affections of the Conjunctiva and Cornea, 40; Treatment of Puerperal Eclampsia—Perchloride of Iron and Magnesia, 41; Carbolic Acid Prepartions, 42; Prescription for Softening of Bones in Clildren—The Sesquichloride of Iron, 43; Congentital Contraction of the Vagina relieved by Gradual Dilitation, by means of Gentian Root—Vaginismus, 44; Compound Syrup of Assafœtida—Benzoating Ointment Extemporaneously, 45; Benzoic Acid Ointment for the Relief of Anul Fistula—Antiseceptic Cere-Cloth, 46; Cholera Infantum—Chilblains, 47; Use of Carbolic Acid to Prevent Pitting After Small-pox Prescription for Juandice—General Neuralgia, 48; Acute Rheumatism—Stimulant Expectorant, 49; Suppurating Scrofulous Glands of the Neck Treated by Drainage—Pleuro Pneumonia, 50.

### EDITORIALS, REVIEWS, ETC.

To Our Subscribers......................................................................52
The Professional Troubles of the Times..................................................54
An Error in Medical Writers.............................................................59
Books and Pamphlets.....................................................................60

